# Characteristics of Esophageal Cancer Among Malawi Natives at a Rural Hospital

**DOI:** 10.7759/cureus.80786

**Published:** 2025-03-18

**Authors:** Joseph Mkandawire, Estifanos B Babulo, Carolyn Moore, Yue Yin, Catherine Lewis, Moses Kasumba

**Affiliations:** 1 General Surgery, Malamulo Adventist Hospital, Makwasa, MWI; 2 Cardiovascular and Thoracic Surgery, Penn Highlands Medical Center, Dubois, USA; 3 Allegheny Singer Research Institute, Allegheny Health Network, Pittsburgh, USA; 4 Surgery, Allegheny Health Network, Pittsburgh, USA

**Keywords:** adenocarcinoma, eastern africa, esophageal cancer, malawi, squamous cell carcinoma

## Abstract

Background: Esophageal cancer (EC) is more prevalent in Malawi as compared to other African countries. Environmental and socioeconomic factors may play a role in the increased incidence of EC in eastern Africa. However, the risk factors are not well defined.

Objective: We sought to determine the characteristics of patients diagnosed with EC in our rural community.

Methods: We conducted a retrospective chart review of patients diagnosed with EC. Age, gender, presence of gastroesophageal reflux disease (GERD), HIV status, and tumor histology type were recorded.

Results: Our study demonstrated that the majority of patients did not use alcohol or tobacco (n = 106 (69.28%) and n = 107 (70.39%), respectively). There was an almost equal distribution of males and females, and the major histologic cell type was squamous cell carcinoma (SCC). There were 149 (82.63%) patients with a history of GERD, and 103 (63.98%) patients had a positive history of HIV.

Conclusions: SCC was the most common histological subtype in our study. Our study demonstrated that alcohol and tobacco use did not impact the type of tissue histology in EC, and the majority of patients had a history of GERD. Further research is needed to further delineate the characteristics of EC in Malawi to determine areas of focus in prevention and treatment.

## Introduction

Esophageal cancer (EC) is the eighth most common cause of malignancy and the sixth leading cause of cancer mortality worldwide [1-4]. There is great geographical variation in the incidence of EC, with increased incidence in the “EC belt” that includes south-central Asia and southern and eastern Africa [5-8]. Discrete areas of Africa, including Uganda, Kenya, Tanzania, Malawi, and portions of South Africa, are disproportionately affected by EC [1,2,9,10]. Malawi has the highest rate of EC [2,8]. In eastern Africa as a whole, EC is the third most common cancer in males and females [11].

Risk factors for EC vary depending on the histologic subtype. Squamous cell carcinoma (SCC) is the histological subtype that comprises at least 90% of all cases, and adenocarcinoma (AC) is the second most common histological subtype [1,12]. Increased alcohol consumption, tobacco use, low intake of fruits and vegetables, and low socioeconomic factors are associated with the development of SCC and AC. A diet rich in pickled vegetables, red meat, and drinking hot beverages has been associated with the development of SCC. Obesity and gastroesophageal reflux disease (GERD) are more common risk factors for the development of AC [7]. Less common risk factors associated with SCC include selenium and zinc deficiency, nitrosamines, opium use, polycyclic aromatic hydrocarbon exposure, Lye disease, Plummer-Vinson syndrome, Chagas disease, a history of certain head and neck cancers, radiation treatment, poor oral hygiene, and family history [13].

There are vast differences in the environment, diet, and socioeconomic factors between African people. Racial and ethnic disparities in EC exist. Eastern Africa has the highest incidence of EC [2,11,14], and Malawi has the highest rate of EC [2,8]. Malamulo Adventist Hospital (MAH) has provided surgical expertise in the treatment of EC. However, the incidence of EC diagnosis and the tumor characteristics are largely unknown. We sought to determine the tumor characteristics of patients diagnosed with EC in a rural community in Malawi.

## Materials and methods

A retrospective chart review was performed at a rural hospital in Malawi of all patients diagnosed with EC from January 2015 to December 2019. Age, gender, risk factors, treatment type, tumor characteristics, and tumor location data were extracted. We included all patients aged 18 and older who were diagnosed with an esophageal malignancy. Patients who underwent upper endoscopy and a tissue biopsy confirming a diagnosis of esophageal carcinoma were included. Patients with symptoms such as heartburn, regurgitation, and chest pain responsive to proton pump inhibitors were considered to have GERD. Patients less than the age of 18 and those who were not treated at the local hospital were excluded. Patients operated on for benign diseases and perforations were also excluded. The study was deemed exempt from local Institutional Review Board (IRB) oversight and received ethical approval from the Malawi National Health Sciences Research Committee (NHSRC). Informed consent was waived in accordance with IRB policies and procedures, and de-identified information was compiled from medical records, imaging, and operative reports.

Descriptive statistics were initially computed. Continuous variables were presented as mean and standard deviation, while categorical variables were presented as frequency and percentage. ANOVA or t-tests were conducted to compare continuous variables between groups. The chi-square test or Fisher’s exact test was used to examine the association between categorical variables. All statistical analyses were conducted via Statistical Analysis System (SAS) 9.4 (SAS Institute Inc., Cary, NC, USA), with an alpha level of 0.05.

## Results

There were 179 patients treated with EC at our institution within the study time period. The average age was 53.3 years (23-82 years). There were 91 (50.84%) males with EC in our study population. The majority of the patients did not use alcohol and did not use tobacco (n = 106 (69.28%) and n = 107 (70.39%), respectively). There were 149 patients (82.63%) with a history of GERD and 103 patients (63.98%) had a positive history of a reactive HIV test. The most common histological tumor type was SCC (n = 174 (97.21%). Table 1 describes the baseline characteristics of all patients.

**Table 1:** Descriptive statistics of demographics and tumor type std: standard deviation; NR: nonreactive; R: reactive; GERD: gastroesophageal reflux disease

Variables	Mean (std)/frequency (%)
Age	53.33 (12.91)
Gender	
Female	88 (49.16)
Male	91 (50.84)
Alcohol use	
No	106 (59.22)
Yes	47 (26.26)
Unknown	26 (14.52)
Tobacco use	
No	107 (59.78)
Yes	45 (25.14)
Unknown	27 (15.08)
GERD	
No	25 (13.97)
Yes	149 (83.24)
Unknown	5 (2.79)
HIV	
NR	58 (32.40)
R	103 (57.54)
Unknown	18 (10.06)
Tumor type	
Adenocarcinoma	4 (2.23)
Small cell carcinoma	1 (0.56)
Squamous cell carcinoma	174 (97.21)

We sought to determine whether there was a correlation between gender, age, alcohol or tobacco use, the presence of GERD, and the presence of HIV in the histological subtype. As shown in Table 2, there was no significant difference between tumor type and various risk factors for EC (p < 0.05).

**Table 2:** Statistical analysis of demographics based on tumor type p < 0.05 was considered statistically significant std: standard deviation; N: number; NR: non-reactive; R: reactive; SCC: squamous cell carcinoma; GERD: gastroesophageal reflux disease

Variable	Tumor type			p-value
	Adenocarcinoma	Small cell carcinoma	SCC	
Age, mean (std)	65.75 (10.56)	65.00 (-)	52.98 (12.84)	0.0973
Gender	N (%)	N (%)	N (%)	0.8072
Female	2 (50.00%)	1 (100%)	85 (48.85%)	
Male	2 (50.00%)	0 (0%)	89 (51.155%)	
Alcohol use	N (%)	N (%)	N (%)	0.3128
No	4 (100%)	0	102 (68.46%)	
Yes	0 (0%)	0	47 (31.54%)	
Tobacco use	N (%)	N (%)	N (%)	0.3194
No	4 (100%)	0	103 (69.59%)	
Yes	0 (0%)	0	45 (30.41%)	
GERD	N (%)	N (%)	N (%)	1.0000
No	0 (0%)	0 (0%)	25 (14.79%)	
Yes	4 (100%)	1 (100%)	144 (85.21%)	
HIV	N (%)	N (%)	N (%)	0.2287
NR	0 (0%)	1 (100%)	57 (36.31%)	
R	3 (100%)	0 (0%)	100 (63.69%)	

The t-test analysis was used to determine if there was an association between age and various risk factors in the development of EC. Table 3 demonstrates non-significant results for age between groups (p < 0.05).

**Table 3:** Comparison of age between groups p < 0.05 was considered statistically significant std: standard deviation; GERD: gastroesophageal reflux disease

Group	Yes, mean (std)	No, mean (std)	p-value
Alcohol use	53.29 (13.42)	52.49 (11.77)	0.7237
Tobacco use	53.11 (13.48)	53.20 (11.51)	0.9696
GERD	50.00 (14.40)	53.96 (12.32)	0.1488
HIV	51.83 (12.97)	54.55 (11.87)	0.1781

## Discussion

Malawi has the highest rate of EC in Eastern Africa [2,8] and EC is the most common cause of cancer mortality [15]. EC is the second most common cancer in men and the third most common cancer in women in Malawi [16]. The mean age in our study was 53.3 years, slightly higher than that reported by Mlombe et al., in which the mean age was 47.5 years [16]. The distribution of males and females in our study was fairly equal (91 (50.84%) and 88 (49.16%), respectively). Our data is inconsistent with that published by Mlombe et al. and Kaimila et al., in which males made up more than 60% of cases of EC [15-17].

The role of alcohol use in the development of EC in East Africa and Malawi is uncertain due to patterns of traditional alcohol production and commercial production. The ESCCAPE case-control study, a multicenter study in Malawi, Kenya, and Tanzania, found that alcohol is a significant contributor to the development of EC, particularly in men [18]. Kaimila et al., in a case-control study conducted in two hospitals in the capital of Malawi, demonstrated that alcohol consumption was not associated with the development of SCC (adjusted odds ratio (OR) 0.7) [15]. This is in agreement with our study in which the majority of the patients did not use alcohol.

Tobacco use overall is relatively low in Malawi [19]. However, tobacco use is a known risk factor for the development of EC. Mlombe et al. noted that 40% of their patients had a smoking history [16]. This is higher than in our study, in which 25.14% of patients reported a history of tobacco use. The ESCCAPE study demonstrated an increased risk of SCC in patients who had ever used tobacco (OR 2.4) [20]. We did not find any significant difference in the tumor type in patients who used tobacco (p = 0.3194).

We identified 174 patients with SCC. Of those, 149 (85.21%) had a history of GERD. Historically, GERD has been shown to be a risk factor for the development of Barrett’s esophagus (BE) and AC of the esophagus, with BE increasing AC risk up to 30-fold [7]. In our study, only four (2.23%) patients had AC. Esophageal AC is mainly a disease in Western Europe and North America [7], and previous studies focusing on risk factors of EC in Malawi did not report any incidence of AC [8]. This may explain the low incidence of AC in our population. Due to financial restraints and cultural practices, patients often present late when they already have obstructive symptoms. Wolf et al. demonstrated that 86% of patients diagnosed with EC in Malawi had obstructive symptoms [21]. Therefore, BE is not frequently diagnosed in our patient population. Also, esophagogastroduodenoscopy is oftentimes not available in rural areas and is often only available to surgeons in regional referral centers [21]. Further research is needed to determine if there are cultural or environmental differences in the risk factors of esophageal AC.

HIV was diagnosed in 103 (57.54%) of our patients with EC. We did not find an association between HIV diagnosis and tissue histology type. Although HIV is prevalent in the patient population, similar to Geßner et al. and Mlombe et al., we did not find an association of HIV with EC [8,16]. EC is not considered an AIDS-defining cancer. However, a retrospective study of the Malawi National Cancer Registry demonstrated that rates of EC have risen along with Kaposi’s sarcoma and HIV [22]. Further studies are needed to determine if there is a link between HIV infection and the development of EC.

There are several limitations to this study. The impact of EC in low-income countries such as Malawi, particularly in rural settings, is difficult to describe due to poor cancer registration systems and under-reporting. The lack of sufficient data collection is ultimately a limitation of the study. Our study was too small to determine any difference between histologic tumor type and risk factors for EC, such as tobacco and alcohol use. Due to the small size of our study, we were also not able to determine if there was an increased risk of EC in patients who may have had a history of* Helicobacter pylori *infection or BE.

## Conclusions

EC is the most common malignancy and a significant cause of death in Malawi. Patients often present late and the risk factors are not clear. A retrospective analysis of all patients at our rural facility demonstrated alcohol and tobacco use did not impact the type of tissue histology in EC. GERD and HIV were common in our patient population but not significantly associated with a specific histologic subtype. Further research and improved data collection are necessary to further describe the impact of EC in African nations.
